# Unveiling the epigenetic impact of vegan vs. omnivorous diets on aging: insights from the Twins Nutrition Study (TwiNS)

**DOI:** 10.1186/s12916-024-03513-w

**Published:** 2024-07-29

**Authors:** Varun B. Dwaraka, Lucia Aronica, Natalia Carreras-Gallo, Jennifer L. Robinson, Tayler Hennings, Matthew M. Carter, Michael J. Corley, Aaron Lin, Logan Turner, Ryan Smith, Tavis L. Mendez, Hannah Went, Emily R. Ebel, Erica D. Sonnenburg, Justin L. Sonnenburg, Christopher D. Gardner

**Affiliations:** 1 TruDiagnostic, Inc, 881 Corporate Dr, Lexington, KY 40503 USA; 2 Stanford Prevention Research Center, Department of Medicine, School of Medicine, Stanford University, 3180 Porter Dr, Palo Alto, Stanford, CA 94305 USA; 3 Seattle Children’s Research Institute, Seattle, WA 98101 USA; 4 Department of Microbiology and Immunology, School of Medicine, Stanford University, Stanford University, Palo Alto, CA USA; 5 Department of Medicine, Division of Infectious Diseases, Weill Cornell Medicine, New York, NY USA; 6 Chan Zuckerberg Biohub, San Francisco, CA USA; 7 Center for Human Microbiome Studies, Stanford University School of Medicine, Stanford, CA USA

**Keywords:** Diet and Nutrition, Epigenetic clocks, Aging, Epigenome-wide association study, Vegan, Omnivore

## Abstract

**Background:**

Geroscience focuses on interventions to mitigate molecular changes associated with aging. Lifestyle modifications, medications, and social factors influence the aging process, yet the complex molecular mechanisms require an in-depth exploration of the epigenetic landscape. The specific epigenetic clock and predictor effects of a vegan diet, compared to an omnivorous diet, remain underexplored despite potential impacts on aging-related outcomes.

**Methods:**

This study examined the impact of an entirely plant-based or healthy omnivorous diet over 8 weeks on blood DNA methylation in paired twins. Various measures of epigenetic age acceleration (PC GrimAge, PC PhenoAge, DunedinPACE) were assessed, along with system-specific effects (Inflammation, Heart, Hormone, Liver, and Metabolic). Methylation surrogates of clinical, metabolite, and protein markers were analyzed to observe diet-specific shifts.

**Results:**

Distinct responses were observed, with the vegan cohort exhibiting significant decreases in overall epigenetic age acceleration, aligning with anti-aging effects of plant-based diets. Diet-specific shifts were noted in the analysis of methylation surrogates, demonstrating the influence of diet on complex trait prediction through DNA methylation markers. An epigenome-wide analysis revealed differentially methylated loci specific to each diet, providing insights into the affected pathways.

**Conclusions:**

This study suggests that a short-term vegan diet is associated with epigenetic age benefits and reduced calorie intake. The use of epigenetic biomarker proxies (EBPs) highlights their potential for assessing dietary impacts and facilitating personalized nutrition strategies for healthy aging. Future research should explore the long-term effects of vegan diets on epigenetic health and overall well-being, considering the importance of proper nutrient supplementation.

**Trial registration:**

Clinicaltrials.gov identifier: NCT05297825

**Supplementary Information:**

The online version contains supplementary material available at 10.1186/s12916-024-03513-w.

## Background

While advances in technology and medicine have allowed the average person to live longer, age-related disease and impairment remain an issue that greatly impacts individuals and healthcare systems. Aging is associated with increases in health care costs and financial stress on social insurance systems [1]. In light of these challenges, the field of geroscience has emerged, proposing interventions aimed at slowing down or reversing the molecular changes that occur with aging. These interventions encompass a wide range of factors, including lifestyle modifications, nutrition, medications, sleep, and social factors, all of which can influence the aging process and potentially delay or prevent the onset of multiple chronic diseases, ultimately extending healthy lifespan [2–4]. Consequently, the exploration of nutritional and dietary recommendations has become an increasingly significant area of research within the broader field of aging, providing insights into how dietary choices can impact the aging process and overall health outcomes.

However, unraveling the intricate molecular mechanisms through which diets influence aging necessitates a deeper understanding of the epigenetic landscape [5]. Epigenetic modifications, such as DNA methylation, have emerged as pivotal regulators of gene expression and provide a promising avenue for investigating the effects of vegan diets on the aging process [6]. The epigenetic effects of a vegan diet, in comparison to an omnivore diet, remain largely unexplored, with limited available evidence. Although certain studies have indicated potential positive impacts of specific components of a vegan diet, such as heightened intake of vegetables and fruits, on epigenetic aging, concerns have been raised regarding potential deficiencies in essential “epi-nutrients” necessary for effective epigenetic regulation [7]. Notably, vitamins and nutrients, including vitamin B12, vitamin B+, choline, vitamin D, omega-3 fatty acids, and zinc, are among the concerns associated with a vegan diet, as their availability may be compromised. Furthermore, other work on diets has aimed to discover the association between diets and longevity [8, 9]. For instance, the Mediterranean diet has been documented to slow the progression of frailty with aging [10]. Dietary protein intake is another important factor considered in aging and frailty, with many studies showing beneficial impacts of protein regardless of animal or plant origin [11]. These and other studies have provided mixed notions of a healthy vegan diet, necessitating additional interrogation of its impact on aging and disease outcomes, as measured by aging markers.

Epigenetic clocks, derived from DNA methylation patterns, have emerged as powerful tools for estimating biological age and predicting age-related outcomes. These clocks have also been refined over time to incorporate known clinical factors, making them sensitive and reliable indicators of aging-related changes [12]. Additionally, epigenetic interpretation algorithms have proven valuable in predicting relative immune cell levels and protein expressions, providing insights into immune system functionality through immune deconvolution [13–15]. Moreover, these clocks can estimate the number of cell cycle divisions, reflecting cellular senescence and potential disease susceptibility [16].

While aging intervention studies face the challenge of requiring sufficiently long periods to show statistically significant effect, advancements in DNAm-based analysis, such as phenotypically and clinically trained DNAm clocks, have allowed for changes in the pace of aging and risk factors related to aging to be studied [17]. Epigenetic age trials using these epigenetic clocks have found that different diets such as a Mediterranean diet and DASH diet have shown improvements of aging pathways and markers, including protective effects of immunosenescence markers, activation of mTOR pathway, and epigenetic aging [18, 19]. In particular, a Mediterranean diet has been shown to both slow aging and delay the onset of frailty [20].

Given the discussion on which diets are most beneficial to longevity, this study aims to identify the effect of an 8-week plant-based or healthy omnivorous diet on blood DNA methylation in twins and evaluate age-related risk factors and health biomarkers. The novelty of this study includes the twin-pair study design which controls for genetic, age, and sex differences, while highlighting the methylation changes based on diet. Furthermore, this is the first study assessing the impact of epigenetic measures on twin-pair study design, and specifically addressed whether diet impacts such measures. Finally, we conducted a differential methylation analysis using the twin-pair design to identify potential DNAm markers which are related to the application of a healthy vegan or omnivorous diet, while also identifying DNAm markers which differentiate between diets. This comprehensive approach will provide insights into how diet type influences epigenetic dynamics and contribute to our understanding of potential interventions in the process of nutrition.

## Methods

### Ethical approval and study design

**Fig. 1:**
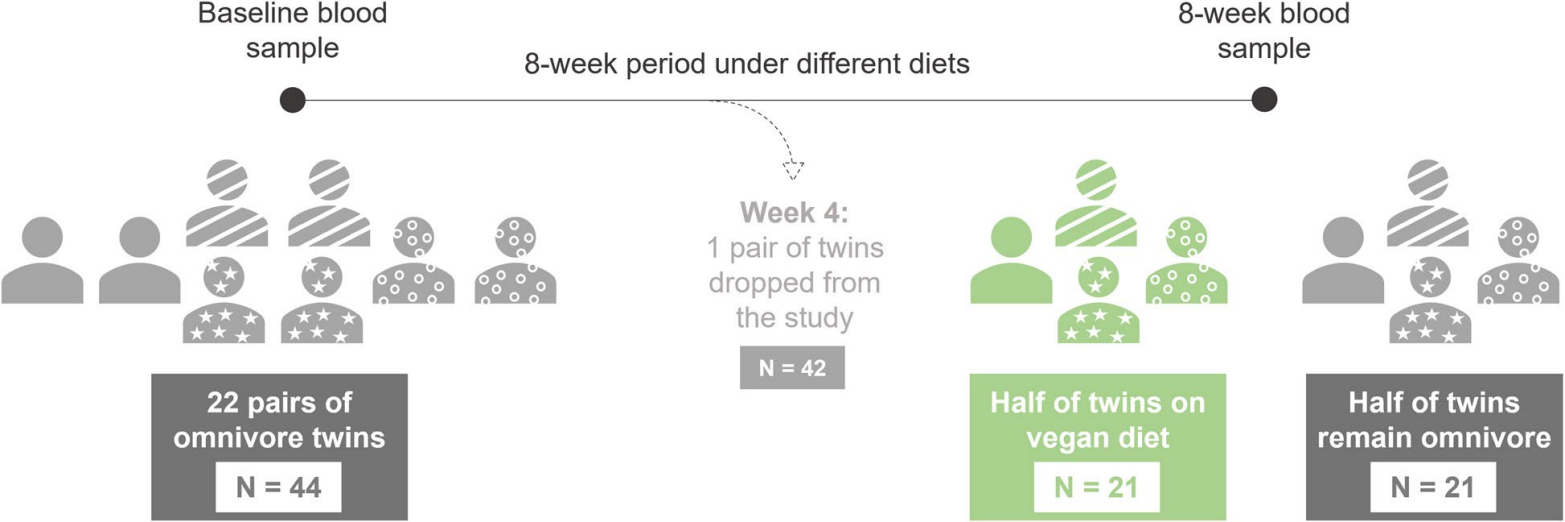
Timeline diagram for the study design. A total of 21 pairs of twins (*N*=42) were subjected to a vegan diet (*N* = 21, labeled in green) and an omnivore diet (*N* = 21, labeled in orange). Blood was collected for baseline at the start of the trial (week 0) and at the end of the trial (week 8) and methylation states were quantified using the EPIC 850k array

Procedures adhered to the ethical standards of the Helsinki Declaration, approved by the Stanford University Human Subjects Committee (IRB protocol 63955, approved March 9, 2022). Written informed consent was obtained from all participants. The study, a single-site, parallel-group dietary intervention trial, randomized generally healthy adult twins to either a healthy vegan or omnivorous diet for 8 weeks. Enrollment commenced in March 2022, concluding in May 2022, with the final follow-up in July 2022. The trial employed the CONSORT reporting guideline for randomized clinical trials, focusing on the primary outcome: the 8-week change in DNA methylation profiles from baseline. Secondary outcomes encompassed triglycerides, HDL-C, glucose, insulin, TMAO, vitamin B12, and body weight, serving as controls for relevant methylation risk scores and were published previously [21]. Diet quality, adherence, and study design are illustrated in Fig. 1.

### Participant recruitment and eligibility

The goal was to recruit 22 pairs of identical twins—controlled for sex, age, and ethnicity—primarily from the Stanford Twin Registry and other twin registries, including Netflix’s pre-recruited participants interested in a documentary on vegan diets. Inclusion criteria involved participants aged ≥18, part of a willing twin pair, with BMI <40, and LDL-C <190 mg/dL. Exclusions included uncontrolled hypertension, metabolic disease, diabetes, cancer, heart/renal/liver disease, pregnancy, lactation, and medication use affecting body weight or energy. Eligibility was determined via online screening, followed by an orientation meeting and in-person clinic visit. Randomization occurred only after completing baseline visits, dietary recalls, and questionnaires for both twins. One twin pair (which started the study, did not abide by the above requirements and thus was removed from the study. Ultimately blood samples from 21 numbers of twin pairs (*N* = 42) were considered for downstream analyses. Full details of the participant profiles are detailed in Landry et al. 2023 [21].

### Dietary intervention and lifestyle changes

The study comprised two 4-week phases: delivered meals and self-provided meals. Trifecta Nutrition supplied meals for the first 4 weeks, tailored to omnivorous and vegan diets. Health educators facilitated nutrition classes via Zoom, emphasizing principles like choosing minimally processed foods and building balanced plates. The omnivorous group received animal product targets (e.g., 6–8 ounces of meat, 1 egg, and 1.5 servings of dairy), while the vegan group avoided all animal products. Dietary intake was assessed through unannounced 24-h recalls and participant logs on the Cronometer app, capturing food intake details at baseline, week 4, and week 8. Dietary data quality was ensured through trained dietitian interviews and app records, used to evaluate diet quality and adherence. To account for lifestyle changes, participants filled out surveys on global health, fatigue, stress, and physical activity, at baseline and week 8. Participants exhibiting notable changes in any of these factors were not considered in the analyses.

### PCR-based telomere estimation

DNA was extracted from whole blood stored at −80 °C with the QIAamp DNA blood mini kit (QIAGEN cat# 51106). Relative telomere length was measured by quantitative polymerase chain reaction (qPCR), expressed as the ratio of telomere to single-copy gene abundance (T/S ratio) [22, 23]. A detailed protocol can be found on the Telomere Research Network’s website (https://trn.tulane.edu/wp-content/uploads/sites/445/2021/07/Lin-qPCR-protocol-01072020.pdf). The inter-assay coefficient of variation (CV) for this study is 2.0%±1.7%. The baseline and follow-up samples of the same participant were processed in the same batch throughout the whole assay. Lab personnel is blind to all the demographic and clinical data.

#### DNA methylation assessment

Whole blood was collected at baseline and at week 8 for DNA methylation preparation and analysis. Majority of twin pairs (20 twin pairs, *N* = 40) were collected as biological replicates per time point and individual using Dried Blood Spot cards; one twin pair (*N*=2 patients) which had triplicate collections in which two were collected by dried blood spot and one using the tasso. Blood collected by the clinics was sent to TruDiagnostic labs in Lexington, KY, for DNA extraction and methylation processing. Using the EZ DNA Methylation kit (Zymo Research), 500 ng of DNA was bisulfite-converted following the manufacturer’s instructions. Bisulfite-converted DNA samples were randomly assigned to wells on the Infinium HumanMethylationEPIC BeadChip, and the subsequent steps included amplification, hybridization, staining, washing, and imaging with the Illumina iScan SQ instrument to acquire raw image intensities. Longitudinal DNA samples for each participant were assessed on the same array to mitigate batch effects. Raw image intensities were saved as IDATs for further processing.

### DNAm data processing

Raw IDATs underwent processing using the *minfi* pipeline [24]. Samples of low quality were identified with ENMix based on variance of internal controls, flagging samples showing more than 3 standard deviations away from the mean control probe value [25]. However, no outlier samples were identified, and thus, all samples were considered for analysis. DNAm normalization involves Gaussian mixed quantile normalization (GMQN) to correct between batch collections and BMIQ normalization to normalize intra-sample variance within chips [26]. Probe-level analysis masked probe sets without at least one intensity fluorescence above the background as implemented by pOOBAH. Missing beta values were imputed using K nearest neighbor (KNN) imputation.

### Deriving estimates of epigenetic clocks and methylation-based metrics

Epigenetic clocks were calculated from cleaned beta values, focusing on clocks like Horvath multi-tissue [27], Horvath skin and blood [28], Hannum [29], PhenoAge [30], GrimAge v1 and v2 [31, 32], and DNAmTL [33]. To ensure that values were highly reproducible, the principal component versions of these clocks were utilized as described by Higgins-Chen et al. [12]. Individual systems clocks were calculated using the framework presented by Sehgal et al. [34]. Clocks were calculated using a custom R script available on Github. DunedinPACE was calculated using a custom script available from Github (https://github.com/danbelsky/DunedinPACE, [35]). Additional non-epigenetic age metrics included relative percentages of 12 immune cell subsets imputed using EpiDISH [15], 116 methylation-based predictions of biochemical and lifestyle risk factors using MethylDetectR [36], and 396 epigenetic biomarker proxies [14]. All epigenetic metrics such as clocks, telomere length, immune deconvolution, EpiScore, and EBPs, were residualized prior to statistical analysis by using the *lmer()* R package as such:

### Residualized epigenetic metric = resid(lmer(Epigenetic predictor ~ Chronological Age + Sex + PC1 + PC2)

Estimates of EpiScores and EBPs were calculated using multivariate models described previously. Briefly, these estimates were derived by modeling DNAm beta values to predict relative protein estimates, as quantified by Olink and SEER/Mass Spec; metabolite estimates, as quantified by the Metabolon panel; clinical values; and clinical and laboratory protein estimates collected from various clinics and panels [14, 37]. Resulting scores and estimates were then used for statistical analyses. All comparisons utilized paired Wilcoxon-rank sum tests faceted by diet type, with significance set at unadjusted *p* < 0.05.

### Assessment of concordance for DNAm and surrogate values

Analyses of telomere and BMI values performed between the reported clinical/qPCR values and DNAm predicted values were conducted in R. Values were scaled using the scale() function prior to comparison. Cohen’s *d* statistics were calculated by inputting scaled values into the cohen.d() function available in the *effsize* library. Statistical significance was assessed using paired Wilcoxon-rank sum tests implemented in the wilcox.test() package. Spearman correlations and associated *p*-values were calculated using the cor.test() package in R and setting the method = “spearman”.

### Differentially methylated analysis

Differential methylation analysis was conducted using processed beta values logit-transformed to M-values with the *BetaValueToMValue* function from the *sesame* R package. No additional probes (e.g., sex associated probes) were pre-filtered in order to prior to the analysis. However, technical variation and sex were considered in the final model for differential methylation. *Limma* package was applied across the four comparisons: within vegan (week 0 vs. week 8), within omnivore (week 0 vs. week 8), cross-sectional Vegan vs. Omnivore (at week 8) and cross-sectional Vegan vs. Omnivore (week 0, or baseline). Differentially methylated loci (DMLs) were identified using different modeling types based on comparison. For within diet comparisons which were longitudinal, multivariate linear models were controlled for fixed effects such as chronological age, BMI, sex, beadchip, 5 immune cell percentages (basophils, CD8T naive, eosinophils, NK, and Neu), the first three principal components of technical probes. For the cross-sectional comparisons, the same fixed effects and PC components were used; however, the individual ID was used in the longitudinal comparison. The inflation or deflation of *P*-values across the methylome was assessed with Q-Q plots and lambda values [38]. DMLs were identified with a significance level of unadjusted *p* < 0.001. False discovery rate (FDR) were also calculated as implemented within the *limma* package and reported.

### GREAT analysis

Functional annotation of DMLs was performed using the GREAT pipeline to identify significant gene ontology terms, as implemented in the *rGREAT* R package [39]. Significant enrichment terms were identified using a Hyper_Raw_PValue < 0.0001; however, only those passing a correct *p*-value (FDR < 0.05) were discussed.

## Results

### Description of study population

To investigate the impact of diet on the methylome, blood samples from a randomized clinical trial were used to quantify methylation [21]. As shown in Fig. 1, to quantify methylation, whole blood was collected to establish a baseline measure of methylation at the time of starting the trial (week 0) and at the conclusion of the study (week 8). Baseline characteristics by diet group appear in Table 1. Among 21 pairs of twins, the randomized mean age was 39.9 (SD 13) years, 77.3% were women, and the mean body mass index was 26 (SD 5). The BMI of both cohorts were largely equivalent due to each group matched to paired-twins with equivalent BMI and genetic makeup (average Vegan BMI = 26.3, average Omnivore BMI = 26.2). The paired-twin design developed here is unique as it controls for genetic and physiological differences between individuals surveyed, which ultimately increases the power of statistical comparisons across the two groups. Full descriptions and characteristics of the study population are detailed previously [21].

**Table 1:** Baseline characteristics of the study participants

	**Vegans**	**Omnivores**
**Sample size (*N*)**	21	21
**Males (%)**	5 (18.5%)	5 (18.5%)
**Females (%)**	16 (76.2%)	16 (76.2%)
**Mean age (SD)**	39.9 (13)	39.9 (13)
**Mean BMI (SD)**	26.3 (5)	26.2 (5)

### Diet type impacts changes in epigenetic age

**Fig. 2:**
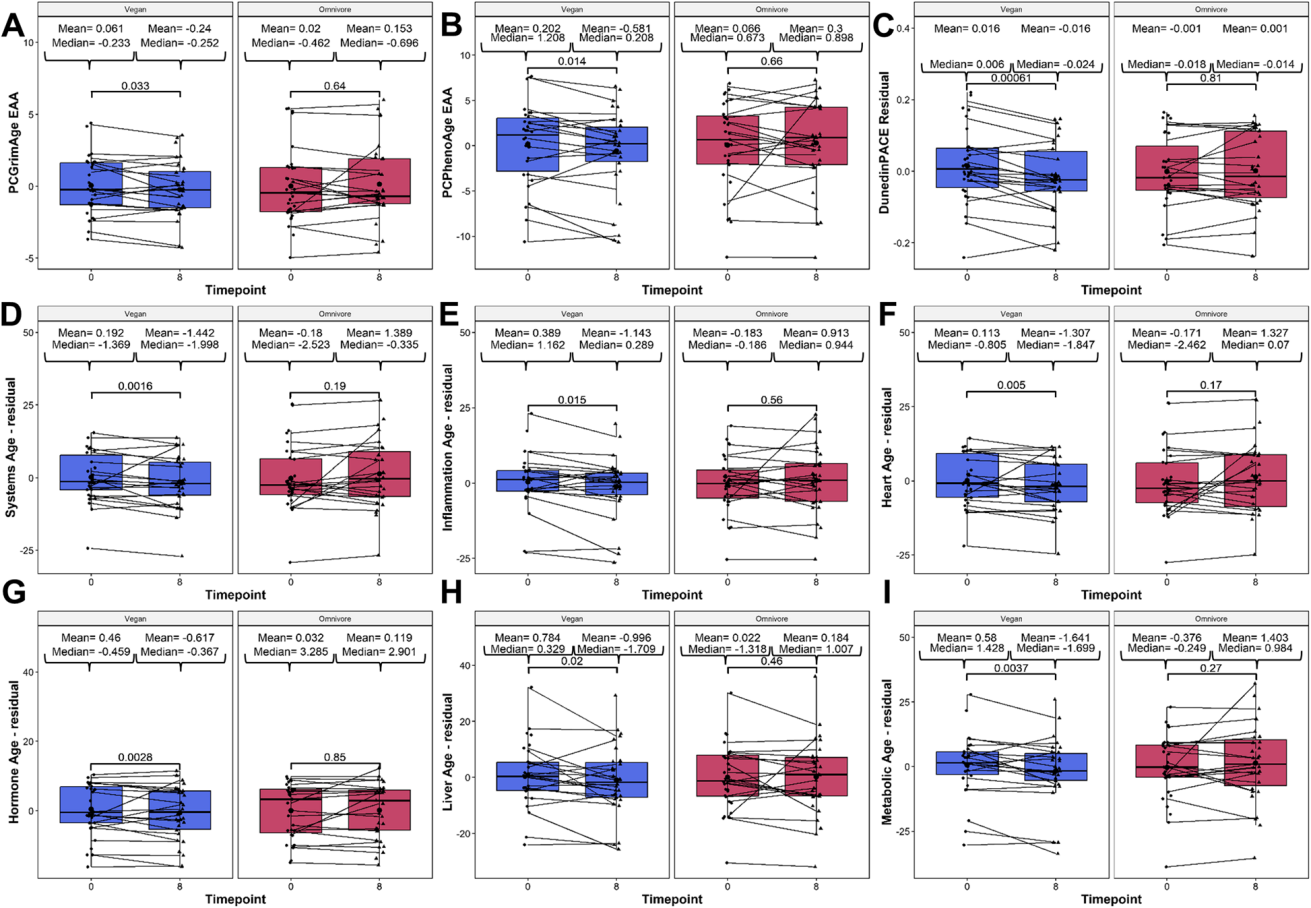
Boxplot showing the evolution of epigenetic age acceleration (EAA)/residuals among the significant epigenetic age clocks and systems-specific clocks based on diet type. Clocks assessed include the **A** PC GrimAge, **B** PC PhenoAge, **C** DunedinPACE, **D** Systems Age, **E** Inflammation Age, **F** Heart Age, **G** Liver Age, **H** Metabolic Age, and **I** Musculoskeletal Age. On the *X*-axis, the time points of measurements in weeks. On the *Y*-axis, the EAA/residual measure. EAA, or residual, is defined as the residual calculated between the raw value regressed upon chronological age, and adjusted by sex, technical principal components 1 and 2. On the top, the mean and median values of the EAA at each time point. The *p*-values of the paired Wilcoxon-rank sums test are also displayed in the plots. Lines that connect both boxplots represent the average of each patient’s tests

To investigate the response to diet on biological age and telomere length, we quantified and analyzed several biological age and telomere length predictors derived from DNAm. These included the principal component (PC)-based clocks: the first-generation multi-tissue Horvath (Horvath1) and skin+blood Horvath (Horvath2); and the second-generation PhenoAge, GrimAge, and DNAm telomere clocks. Additionally, several non-PC clocks were included as well: the first-generation Zhang clock based on the elastic net (Zhang-EN) and BLUP (Zhang-BLUP) method; the second-generation multi-omic informed OMICmAge, and the third generation DunedinPACE clock. To better understand the impact of diet on the epigenetic age of specific organ systems, we also calculated the individual ages of 11 organ systems: Heart, Lung, Kidney, Liver, Brain, Immune, Inflammatory, Blood, Musculoskeletal, Hormone, and Metabolic. In addition, a composite age of the system was also calculated as Systems Age. In the vegan group, we observed significant decreases in the following epigenetic age metrics: PC GrimAge (mean Δ EAA = −0.3011, *p* = 0.033), PC PhenoAge (mean ΔEAA = −0.7824, *p* = 0.014), and DunedinPACE (mean Δ PACE residual = −0.0312, *p* = 0.00061) significantly decreased at 8 weeks relative to 0 weeks (Fig. 2A–C). Similarly, we observed significant reductions in the composite systems age metric, which was corroborated by significant reductions of 5 out of 11 systems: Inflammation, Heart, Hormone, Liver, and Metabolic (F igure 2D–I). In contrast, no epigenetic clock or telomere measure exhibited significant changes in the omnivorous cohort, suggesting that the omnivorous diet did not induce any epigenetic age methylation changes. Taken together, these findings suggest that the observed DNA methylation changes may contribute to the overall decreases in epigenetic age in response to a vegan diet, which is not observed among omnivores.

### Telomere length quantified by Telomere Shortening Rate (TSR) exhibits changes, but not epigenetic telomere length

We next sought to elucidate the potential impact of vegan diets on telomere length. First, we established an understanding of concordance between estimated telomere length using quantitative polymerase chain reaction (qPCR) and the estimated PC DNAmTL values by quantifying the correlations between the values of all samples, and assessing significant differences agnostic to groups. Overall, we observed an overall correlation > 0.5 between the scaled qPCR and PC DNAmTL values calculated (*⍴* = 0.564; *p* < 1.22e−7), and no significant differences (Wilcoxon-rank sum *p* = 0.9427). This was further supported by a negligible effect size difference between the scaled values (Cohen’s *d* estimate = −2.45e−15, 95% CI = −0.321–0.321). These results suggest that the values generated by both methods are comparable.

**Fig. 3:**
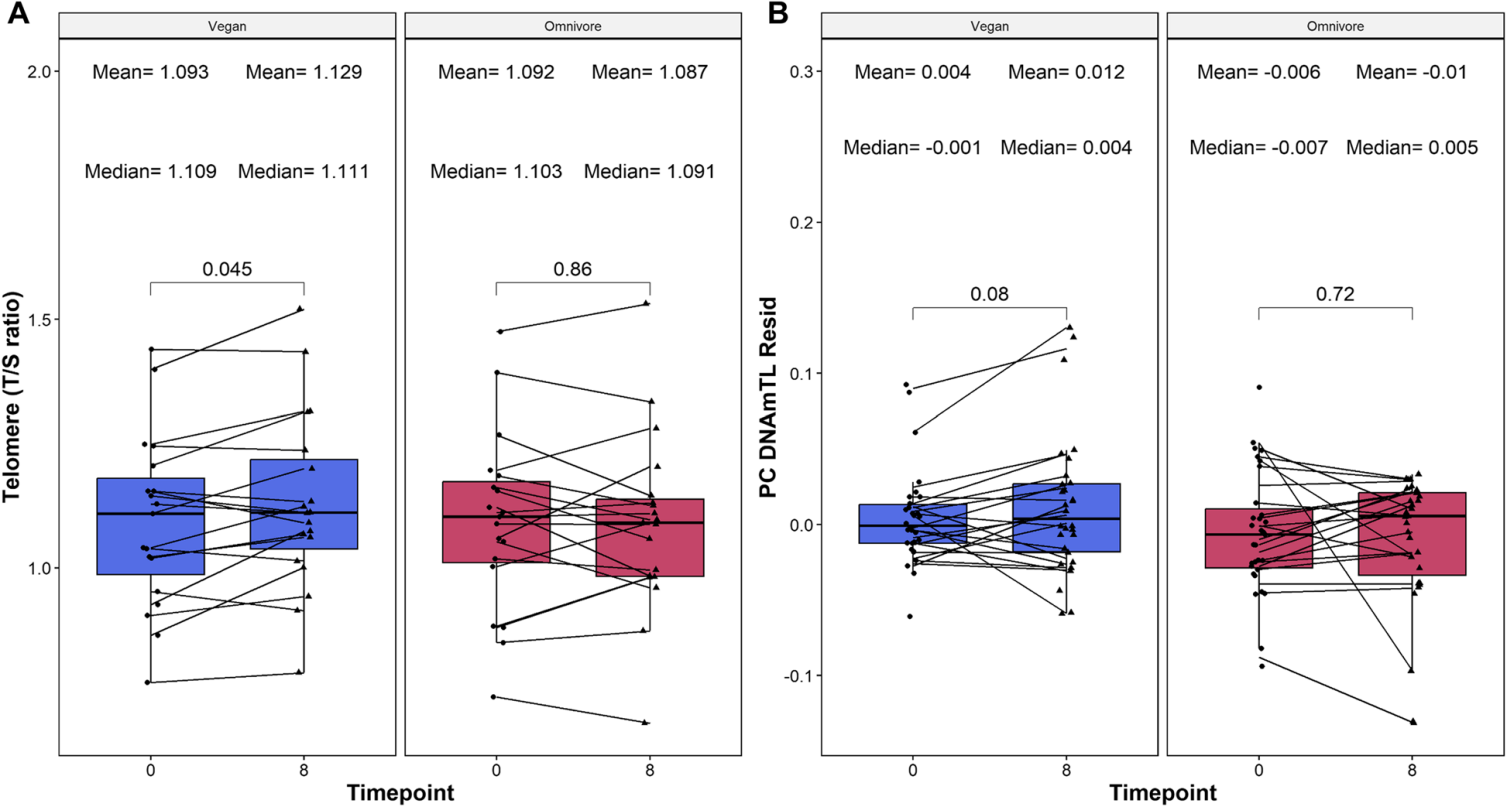
Boxplot showing the change between relative telomere levels as quantified by qPCR and DNA methylation (PC DNAmTL). Telomere qPCR value is reported in panel **A**, while the PC DNAmTL values are reported in **B**. On the *X*-axis, the time points of measurements in weeks. On the *Y*-axis, the T/S ratio is shown for qPCR, or the residual of PC DNAmTL. The PC DNAmTL residual is defined as the residual calculated between the raw PC DNAmTL value regressed upon chronological age, and adjusted by sex, technical principal components 1 and 2. On the top, the mean and medians of the *Y*-axis values at each time point are reported. The *p*-values of the paired Wilcoxon-rank sums test are also displayed in the plots. Lines that connect both boxplots represent the average of each patient’s tests

Next, changes in TSR were assessed between timepoints within each diet among the TSR data. The average telomere length was significantly longer at week 8 than at week 0 for Vegans (*p* = 0.045, Δ T/S ratio = 0.0361, Fig. 3A) but not for omnivores (*p* = 0.86, Δ T/S ratio = −0.0045, Fig. 3A). Furthermore, paired analyses comparing twins between diets within each time point were conducted among the TSR samples, which found that the Vegan group had significantly longer telomeres than their Omnivore twins at week 8 (*p* = 0.01, Δ T/S ratio = 0.042) but not at week 0 (*p* = 0.54, Δ T/S ratio = 0.0013), further confirming that the telomere extension was specific to the vegan diet. This contrasted the findings observed among the PC DNAmTL values, which showed that there were no significant changes between week 8 and week 0 measures among the Vegan or Omnivore cohort (Fig. 3B).

### Analysis of cell cycle changes shows no significant changes based on diet

We next assessed whether diet type exhibited differences in overall mitotic rate as quantified by the mitotic clock output. Using the *epiTOC2* algorithm, we observed no significant changes in either the vegan or omnivore diet when assessing the total number of stem cell replication cycles estimated (*tnsc*) or the intrinsic stem cell cycle rate based on tissue (*irS*). This suggests that diet type did not have an impact on the overall mitotic clock values from the data.

### Vegan diets exhibit significant changes in relative basophil levels

**Fig. 4:**
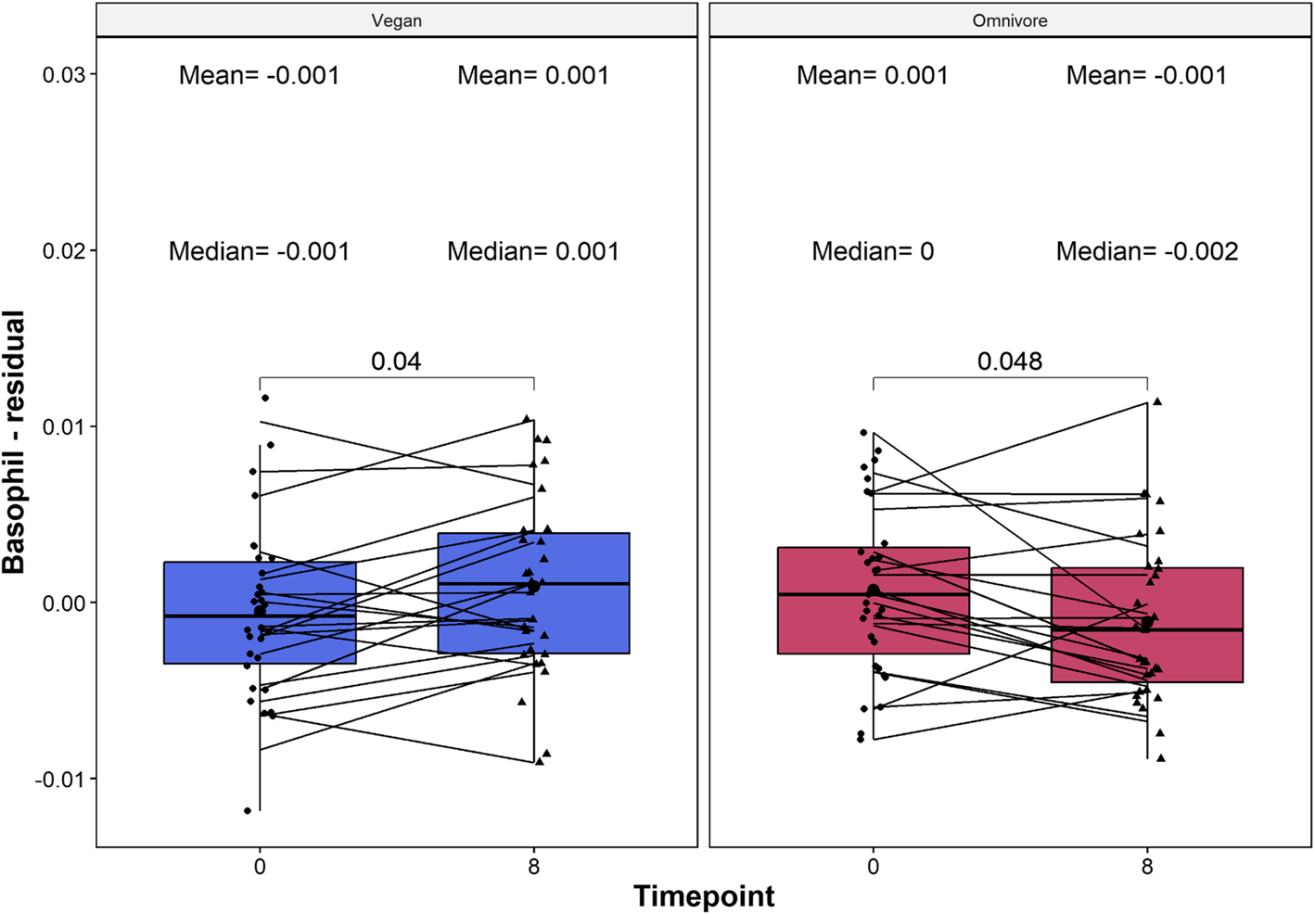
Boxplots showing the evolution of basophil cell subset percentages based on diet type. On the *X*-axis, the time points of measurements in weeks. On the *Y*-axis, the basophil measure. The basophil measure is residualized, which is defined as the residual of the raw deconvolution value regressed upon chronological age, and adjusted by sex, technical principal components 1 and 2. On the top, the mean and median values of the residual at each time point. The *p*-values of the paired Wilcoxon-rank sums test are also displayed in the plots. Lines that connect both boxplots represent the average of each patient’s tests

The immune system undergoes distinctive changes based on dietary choices, with vegan and omnivore diets influencing immune cell behavior in unique ways. Exploring this interplay provides valuable insights into the intricate relationship between diet and the body’s immune defenses. To investigate the impact of diet on the immune system, we next analyzed relative immune cell subset changes throughout the trial among 12 immune cell subsets quantified by the EPIDISH frame: CD8T-naive, CD8T-memory, CD4T-naive, CD4T-memory, basophils, B naive, B memory, T-regulatory, monocytes, neutrophils, natural killer, and eosinophils. We observed significant changes in basophil levels in the vegan and omnivore diets. However, the basophil levels increased in the vegan group (Δ mean = 0.0014, *p* = 0.04, Fig. 4) and decreased in the omnivore group (Δ mean = −0.0018, *p* = 0.048).

### Assessment of type 2 diabetes risk based on loci

**Fig. 5:**
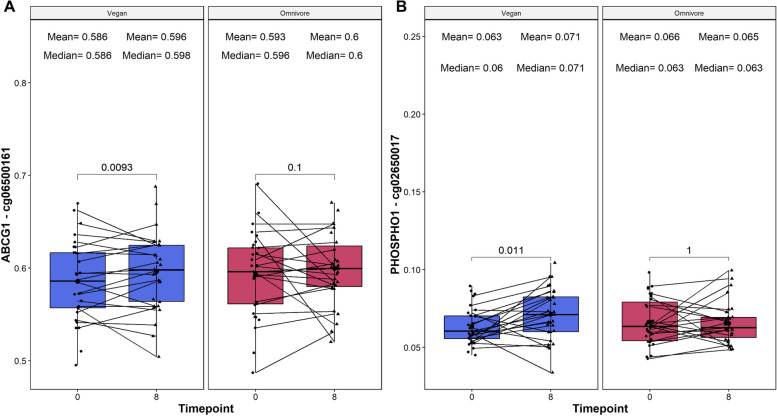
Boxplots showing the relative beta value change of two weight loss methylation sites on the ABCG1 gene (reported on the left) and PHOSPHO1 gene (reported on the right). On the *X*-axis, the time points of measurements in weeks, and the loci beta value which is reported on the *Y*-axis. On the top, the mean and median beta values of the loci at each time point. The *p*-values of the paired Wilcoxon-rank sums test are also displayed in the plots. Lines that are connecting both boxplots represent the average of each patient’s tests

Previous studies have shown plant-based diets associated with reduced type 2 diabetes risk [40, 41]. To test whether epigenetic changes are consistent with previous findings, we analyzed two DNA methylation loci, ABCG1 (cg06500161) and PHOSPHO1 (cg02650017), which are implicated in predicting T2D risk [42]; increased methylation in ABCG1 correlates with a higher T2D risk, while heightened PHOSPHO1 methylation is linked to a reduced risk. In our study, the vegan group displayed a significant increase in methylation at the ABCG1 loci (Δ beta value mean = 0.0105, *p* = 0.0093, Fig. 5A), indicating a potentially elevated T2D risk. Concurrently, an increase in PHOSPHO1 cg02650017 methylation (Δ beta value mean = 0.0079, *p* = 0.011, Fig. 5B) suggests a decreased T2D risk for the vegan cohort. This dichotomy in methylation changes for the two loci within the vegan group underscores a complex relationship between diet and T2D biomarkers, necessitating further investigation for a comprehensive understanding. None of these CpG sites were differentially methylated over time in the omnivore group.

### Analysis of EpiScore markers

Recent efforts have expanded DNA methylation proxies to predict proteins, complex behavioral, and physiological traits [13, 14, 26]. To this end, we utilized these DNAm-based surrogate markers to assess relative changes in response to diet type (Additional file 1: Table S1). In an initial analysis, we utilized EpiScore models previously described: multivariable linear models of beta values used to predict the estimates of the 116 modeled proteins, behavioral and physiological traits [26]. Comparison of EpiScore values between time 8 and time 0 samples detected significant changes in seven EpiScores in the Vegan group, using a unadjusted *p*<0.05: CCL21, MMP1, ENPP7, Testican 2, ADAMTS, CD163, and MMP2. Notably, these EpiScores were not evident in the omnivore group analysis, underscoring diet-specific variations. Conversely, in the omnivore-specific analysis, six EpiScores—Ectodysplasin A, PAPP-A, VEGFA, HGF, Body Fat %, and TNFRSF17—exhibited exclusive significant change at an unadjusted threshold of *p* < 0.05. However, it must be noted that in both analyses, none of the EpiScore values met a multi-comparison corrected significance threshold (adjusted BH, *p* < 0.05). In summary, the methylation-based surrogate markers of complex physiological and behavioral traits identified here suggest that while common markers are present, the majority of changes among the EpiScore are unique among diet types.

### Analysis of Epigenetic Biomarker Proxies (EBP)

We also assessed changes in EBPs: DNAm proxy scores of metabolites, proteins, and clinical values estimated using multivariate linear models composed of DNA methylation values that were previously described [14]. Of the 396, we identified a total of 76 and 89 EBPs which showed significant changes among the vegans and omnivores, respectively, using an unadjusted *p* < 0.05 (Additional file 2: Table S2). After correcting for multiple comparisons (BH < 0.05), 13 and 19 EBPs satisfied the adjusted threshold. In the following independent analyses were performed between vegan and omnivore diets respectively to identify EBPs which showed (1) unique changes among diet types, (2) consistent changes among diet types, and (3) opposing changes among diet types.

We identified 33 EBPs that showed uniquely significant changes (unadjusted *p* < 0.05) among the vegan cohort: *androsterone glucuronide*, *homovanillate (HVA)*, *branched-chain*, *straight-chain*, or cyclopropyl 10:1 fatty acid (2)*, *Liver albumin*, *CCL18*, *PON1*, *dehydroepiandrosterone sulfate (DHEA-S)*, *PON1*, *glutamine_degradant*, *leucine*,* 1*,*5-anhydroglucitol (1,5-AG)*, *CRP*, *arabitol/xylitol*, *retinol (vitamin A)*, *3-hydroxyindolin-2-one sulfate*, *2-methylcitrate/homocitrate*, *deoxycholic acid glucuronide*, *7-hydroxyindole sulfate*, *alpha-CMBHC glucuronide*, *PCOC1*, *riboflavin (vitamin B2)*, *1-palmitoyl-GPC (16:0)*, *PCOC1*, *GRN*, *S-carboxyethylcysteine*, *FETUA*, *CSPG2*, *dimethyl sulfone*, *carotene diol (2)*, *guanidinosuccinate*, *6-oxopiperidine-2-carboxylate*. Among these, 3 EBPs - *androsterone glucuronide*, *homovanillate (HVA)*, *branched-chain*, *straight-chain*, or cyclopropyl 10:1 fatty acid (2)* - further passed an adjusted p-value threshold (BH < 0.05), suggesting that the EBPs identified here represent potential biomarkers uniquely altered in response to a vegan diet at 8 weeks.

Among omnivores, we observed 46 EBPs which showed significant changes only among the omnivore diet cohort: *4-methoxyphenol sulfate*, *N-methylpipecolate*, *N-acetylcitrulline*, *sucrose*, *vanillactate*, *uridine*, *N-acetyltyrosine*, *3-hydroxybutyroylglycine*, *Liver_ALP*, *tryptophan*, *dihydroferulic acid sulfate*, salicyluric glucuronide*, *picolinate*,* 3,5-dichloro-2,6-dihydroxybenzoic acid*, *urea*, *galactonate*, *thyroxine*, *2-acetamidophenol sulfate*, *cystathionine*, *sphinganine-1-phosphate*, *choline phosphate*, *picolinoylglycine*,* N,N,N-trimethyl-5-aminovalerate*, 1-pentadecanoyl-GPC (15:0)*, *TLL1*, *PCOC1*, *glycochenodeoxycholate 3-sulfate*, *trans-4-hydroxyproline*, *gentisate*, *catechol glucuronide*, *citramalate*, *ferulic acid 4-sulfate*, *PLMN*, *sedoheptulose*, *vanillic acid glycine*, *PCOC1*, *BMP1*, linoleoylcarnitine (C18:2)*, *1-methylguanidine*, *isobutyrylcarnitine (C4)*, *indolebutyrate*, *hypoxanthine*, *Smoking_PackYears*, *3-hydroxyoctanoylcarnitine (1)*, eicosenoylcarnitine (C20:1)*, and *BMP1*. Among these, 8 EBPs passed an adjusted *p*-value threshold (BH < 0.05), with *4-methoxyphenol sulfate*, *N-methylpipecolate*, *N-acetylcitrulline*, *sucrose*, *vanillactate*, *uridine*, and *N-acetyltyrosine* exhibiting a significant increase among the omnivore group at week 8, and a significant decrease in *uridine* and *3-hydroxybutyroylglycine*. These EBPs represent biomarkers uniquely associated with the omnivore diet but not vegan diet.

We also identified several EBPs which showed consistent changes among the diet types. Approximately 16 of the vegan EBPs showed significant increase in both vegan and omnivore diet types which included CCL16, glucuronide of C12H22O4 (2)*, 2-methoxyhydroquinone sulfate (1), adenosine, *lactosyl-N-palmitoyl-sphingosine (d18:1/16:0)*, 1-stearoyl-2-dihomo-linolenoyl-GPC (18:0/20:3n3 or 6)*, *N-acetylalliin*, *N-carbamoylalanine*, *caffeine*, *carnitine*, 1-palmitoyl-2-arachidonoyl-GPE (16:0/20:4)*, *FETUA*, *2,3-dihydroxy-2-methylbutyrate*, *LYSC*, eicosenedioate (C20:1-DC)*, and *1-methyl-5-imidazoleacetate)*. Conversely, approximately 21 exhibited decreases among both diets, which included *10-undecenoate (11:1n1)*, *1,2-dipalmitoyl-GPC (16:0/16:0)*, *3-carboxy-4-methyl-5-propyl-2-furanpropanoate (CMPF)*, *salicylate*, *succinylcarnitine (C4-DC)*, 1-margaroyl-2-arachidonoyl-GPC (17:0/20:4)*, *5-methyluridine (ribothymidine)*, *Glucose*, *2-aminoheptanoate*, *stearoyl-arachidonoyl-glycerol (18:0/20:4) *[1]*, PCOC1, *proline*, *ibuprofen*, *11-ketoetiocholanolone glucuronide*, *homoarginine*, *Triglyceride*, *PCOC1*, *PCOC1*, 1-stearoyl-2-adrenoyl-GPC (18:0/22:4)*, *BMI*, and *3-hydroxyphenylacetoylglutamine*). These EBPs represent surrogate markers of metabolite, clinical, and proteins which changed regardless of diet type, suggesting these as non-diet associated EBP markers.

However, we observed 6 EBPs which showed opposing changes in EBP levels: *serine*, 1-margaroyl-GPE(17:0)*, and *4-acetamidophenol*, showed significant increases among vegans, and significant decreases among omnivores, while *ergothioneine*, *indoleacetylglutamine*, and *creatinine* showed a significant decrease among vegans compared to the increase observed among omnivores. The significant, and opposing, changes between diets suggest that these represent diet-based interactions significant in one diet but not the other.

### Assessing congruence among BMI and BMI-EBP measures

**Fig. 6:**
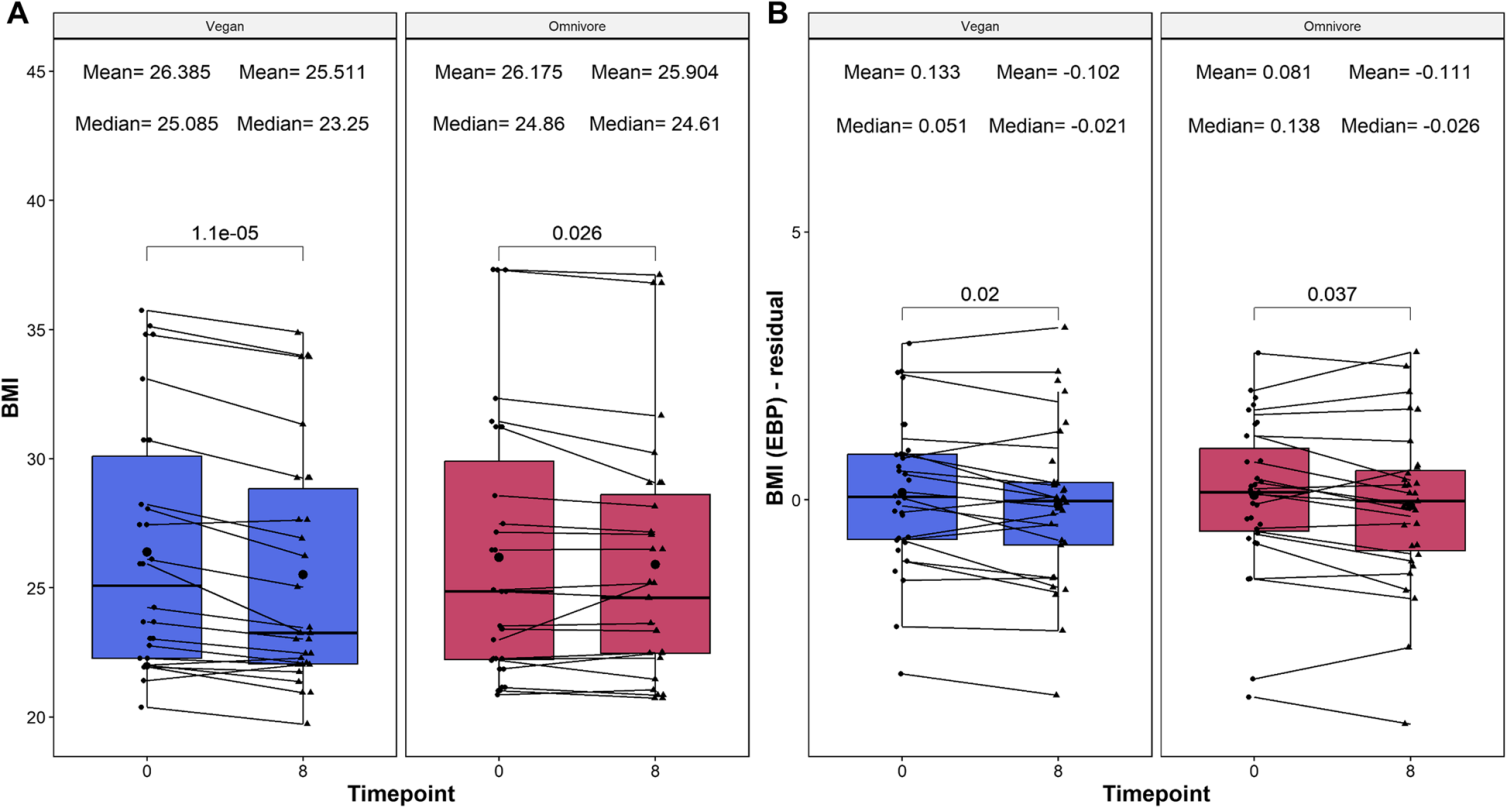
Boxplot showing the evolution of BMI values calculated from clinical measures (reported on the left) and epigenetic biomarker proxy (EBP) measures (reported on the right). On the *X*-axis, the time points of measurements in weeks. On the *Y*-axis, the BMI measure. The BMI-EBP measurements are reported as residuals, which are defined as the residual of the raw BMI value regressed upon chronological age, and adjusted by sex, technical principal components 1 and 2. No residual calculation was done for the clinical EBP. On the top, the mean and median values of the BMI at each time point. The *p*-values of the paired Wilcoxon-rank sums test are also displayed in the plots. Lines that connect both boxplots represent the average of each patient’s tests

To better assess the reproducibility of the EBPs calculating clinical measures, we compared the BMI-EBP changes relative to the BMI-clinical values that were collected within this study. First, we assessed the correlation between all BMI-clinical values with the BMI-EBP counterparts, which resulted in significant correlations among the values (*⍴* = 0.275, *p* = 0.0022) and negligible difference in mean difference (Cohen’s *d* = 1.10e−16, 95% CI = −0.252–0.252) after scaling. Analysis of the longitudinal data identified that both BMI measurements showed consistent significant decreases in both diet types (*p* < 0.05). However, the magnitude of change was higher in the BMI-clinical values compared to the BMI-EBP values (Fig. 6). Taken together, these findings exhibit the reproducibility of the BMI metrics among the EBPs relative to their clinical counterparts.

### Global EWAS analysis identifies epigenetic markers of vegan and omnivorous diets

We utilized an exploratory epigenome-wide analysis approach of 866,836 CpGs to identify candidate differentially methylated loci associated with a vegan or omnivore diet. To run the correct EWAS model for each comparison, we first tested for test-statistic inflation (lambda) with each EWAS model adjusted by different fixed effects [27]. The final models ultimately chosen reported lambdas closest to 1 in each of the comparison: within vegan (week 0 vs week 8) lambda chosen is 0.97; within omnivore (week 0 vs week 8) lambda chosen is 0.89; cross-sectional week 8 comparison lambda chosen is 1.03; and cross-sectional week 0 comparison chosen is 1.06.

**Fig. 7:**
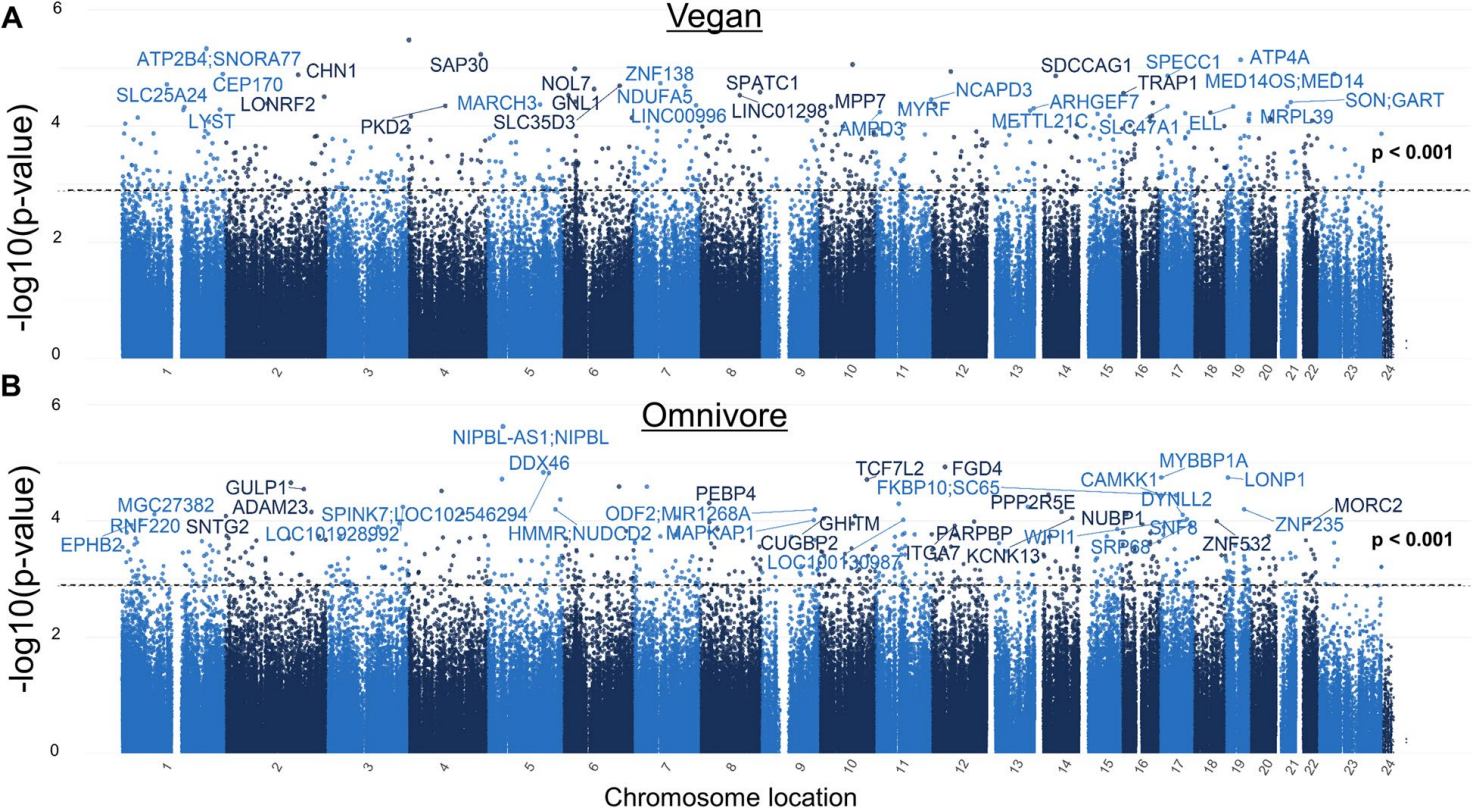
Manhattan plots for the vegan and the omnivore epigenome-wide association studies. The Manhattan plot illustrates genes associated with CpG sites identified in the **A** vegan and **B** omnivore comparison, with each dot representing a CpG site and its vertical position corresponding to the negative logarithm (base 10) of the unadjusted *p*-value for DNA methylation association (significance set at *p* = 0.001). The *x*-axis denotes genomic positions organized by chromosomes, with color-coded dots indicating specific chromosomes, and prominently peaked dots represent significantly associated CpG sites surpassing the genome-wide significance threshold

Utilizing the optimal EWAS models, differentially methylated loci DMLs were identified. In the first comparison, we identified a total of 607 differentially methylated loci (DMLs) associated with 8 weeks of a vegan diet (*p*-value < 0.001) compared to week 0 (Fig. 7A). Among these vegan-diet associated loci, 322 CpG sites showed hypomethylation at 8 weeks, and 312 loci exhibited hypermethylation at week 8. Among the omnivore cohort, a total of 494 DMLs were associated with 8 weeks of an omnivore diet (*p*-value < 0.001) (Fig. 7B), in which 309 CpGs showed increases in DNA methylation and 185 CpGs exhibited loss in DNA methylation at week 8. The full list of DMLs associated with 8 weeks of a vegan or omnivore diet is listed in Additional file 3: Tables S3 and S4 for both analyses. The DMLs identified here represent potential methylation markers of specific dietary interventions in response to the consumption of vegan diet or omnivorous diet, respectively.

**Fig. 8:**
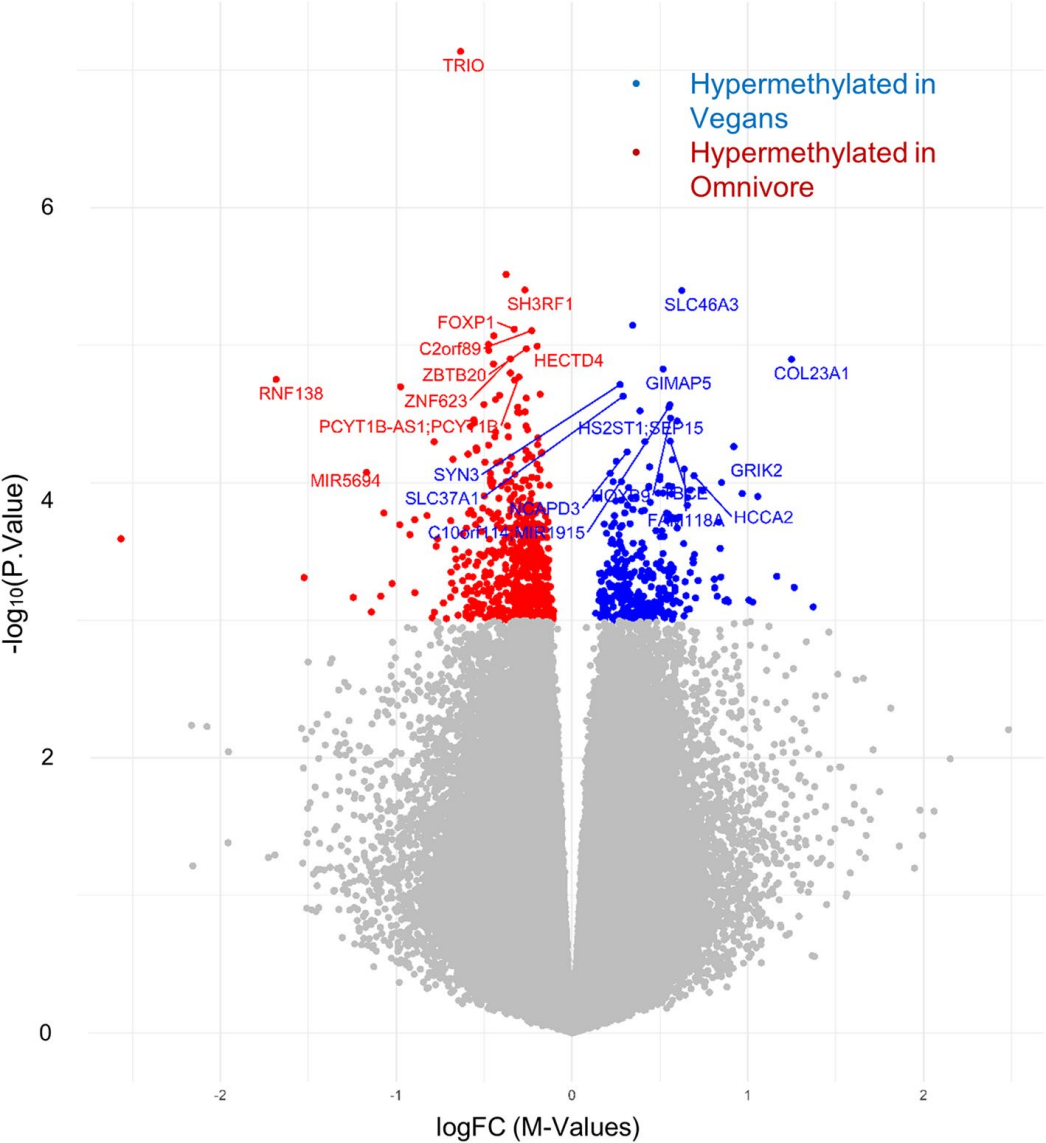
Volcano plot of DMLs identified in the comparison between vegan and omnivore diet at the week 8 time point. The volcano plot illustrates DMLs identified in the Vegan vs. Omnivore comparison, with each dot representing a CpG site and its vertical position corresponding to the negative logarithm (base 10) of the unadjusted *p*-value for DNA methylation association. The *x*-axis denotes the relative log fold change (logFC) of the *m*-values between the vegan and the omnivore diets. Values greater than 0 represent CpGs with greater methylation among vegans (blue), compared to the negative values which represent greater methylation among omnivores (red)

To better understand the specific DNA methylation patterns that differentiated vegan diet samples and omnivorous diets, a cross-sectional analysis comparing these groups at the week 8 time points was conducted. We identified a total of 980 DMLs that were differentially methylated between the participants on an omnivore diet at week 8 and the participants on a vegan diet at week 8 (*p*-value < 0.001). Of the DMLs identified, 317 exhibited hypermethylation in the week 8 vegan samples relative to the week 8 omnivore samples, while 663 DMLs exhibited hypomethylation in the week 8 vegan sample (or greater methylation in the omnivore group) (Fig. 8, Additional file 3: Table S5). Similarly, a cross-sectional analysis at week 0 was also conducted to identify the base difference in methylation between the vegan and omnivore twins at the time of starting the trial. A total of 834 DMLs were identified between the diets at week 0, with 385 hypermethylated loci in the vegan samples compared to the omnivores (average logFC difference of 0.498), and 452 hypomethylated DMLs (or greater in omnivores compared to vegans) exhibiting an average logFC difference of −0.355. Baseline DMLs represent methylation differences of twins at their base and are reported in Additional file 3: Table S6.

Finally, to answer if the CpGs identified at week 8 were uniquely differentially methylated to the identified week 0 DMLs, we compared the cross-sectional comparison lists. Only 2 CpGs (cg04227789, cg18301717), or 0.2%, overlapped in both comparisons, suggesting that the DMLs identified at week 8 are likely due to a diet-based effect.

### Gene ontology pathway analysis of diet-associated DMLs

To better understand the transcriptionally relevant biological processes associated with methylation changes, gene ontology (GO) enrichment analyses were conducted among DMLs identified in the week 8 vegan and omnivore comparison. To ensure transcriptional relevance, DMLs were inputted into the GREAT software by direction of methylation and CpGs overlapping with cis-regulatory or other regulatory regions were linked to genes and thus assessed for their relationship to various biological processes (BP), molecular function (MF), and cellular component (CC) associations. Significantly hypermethylated DMLs in the vegan group, or hypomethylated among omnivores, were reported as significantly enriched for GO-BP terms such as *paracrine signaling*, *response to beta-amyloid*, *neuron apoptosis*, and several developmental GO-BP terms (adjusted BH *p*-value < 0.05). In addition, molecular function (GO-MF) terms such as *Ras guanyl-nucleotide exchange factor activity* were enriched for sites that exhibited significant hypermethylation among the vegans, and lower among the omnivores (adjusted BH *p*-value < 0.05, Additional file 4: Table S7). CpGs that were hypermethylated among omnivores, and hypomethylated among vegans, were enriched for GO-BP terms associated with cell cycle (negative regulation of the G0-G1 transition), genomic imprinting (*regulation of gene expression by genetic imprinting*), *cytosolic calcium ion transport*, and *cellular response to alcohol.* Cell cycle and transcriptional activity were further supported by the enrichment of GO-MF terms associated with RNA polymerase activity and transcriptional processes (*protein phosphatase inhibitor activity*, *RNA polymerase II regulatory region DNA binding*, and *promoter-specific chromatin binding*). Full results for biological associations to CpGs differentially methylated between diet types are listed in Additional file 4: Table S8. In summary, the significant gene ontology terms identified reveal distinct associations with key biological processes and molecular functions, shedding light on the epigenetic mechanisms altered in response to dietary choices.

## Discussion

In this study, we sought to elucidate the impact of a “healthy vegan” or a “healthy omnivorous diet” on epigenetic age, telomere length, immune cell subsets, and type 2 diabetes (T2D) risk-associated CpGs, building on current knowledge of nutrition on both diets. Our findings reveal distinct responses to vegan and omnivore diets, aligning with existing literature on the subject. Notably, the vegan cohort exhibited a significant decrease in epigenetic age acceleration, as demonstrated by reductions in multiple epigenetic aging clocks, all of which were trained upon clinical [28] and phenotypic scores (PC GrimAge, PC PhenoAge, 28-31). The usage of systems-specific aging predictors further specified which organ systems showed age improvements, resolving five specific systems that showed aging improvements among the vegan cohort and not omnivores. These findings are consistent with previous research highlighting the potential anti-aging effects of plant-based diets, known for their rich antioxidant content and anti-inflammatory properties [43–45]. However, the significant impact of basophils in the vegan group contrasts with studies emphasizing the immunomodulatory benefits of plant-based diets, suggesting that further investigation into the nuanced interactions is warranted [46]. These comprehensive findings underscore the complex interplay between diet, epigenetic regulation, immune function, and metabolic health, offering valuable insights for future research and personalized health interventions.

The measures investigated in our study offer a holistic perspective on biological aging without isolating system-specific aging processes, as highlighted by Ahadi in 2020 [47]. However, the incorporation of the systems age clock in our research addresses this limitation by providing valuable, system-specific insights into aging changes [34]. Notably, our findings reveal significant reductions in key system-specific disease processes, including inflammation, heart, liver, metabolic, and hormonal systems. This nuanced approach aligns with previous research demonstrating that vegan and plant-based diets are associated with lower levels of inflammatory markers [46], lower risk of cardiovascular diseases [48, 49], reduced risk of non-alcoholic fatty liver disease (NAFLD) [50], improve glycemic control and other metabolic factors in individuals with type 2 diabetes [51], and regulated hormonal level outputs in responses such as hot flashes [52]. This approach allows for a more comprehensive understanding of the impact of the studied interventions on specific facets of aging, shedding light on potential areas of targeted intervention for promoting overall health and longevity. The identification of these system-specific changes contributes to a more nuanced and actionable comprehension of the aging process, underscoring the significance of our results in advancing our knowledge of interventions that may influence distinct physiological systems and enhance overall well-being.

One notable difference we observed was the magnitude of telomere length change within the vegan diet, where qPCR-TL analysis identified a statistically significant increase in telomere length, while PC DNAmTL exhibited an insignificant increase. While this finding is consistent with previous investigations that have reported mixed congruency between qPCR-TL and PC DNAmTL values [53–55], our assessment of telomere congruency between the two methods showed moderate correlation (*⍴* > 0.56) and no significant difference between PC DNAmTL and qPCR-TL, as evidenced by the Wilcoxon-rank sum and Cohen’s *d* tests. This suggests that the incongruency between the telomere length changes observed between the two methods could be attributed more to the different signals of telomere biology captured, such as telomere maintenance mechanisms and not telomere length [54].

While there is no gold standard measure of biological aging [56], we analyzed several measures that represent the current DNAm predictors of biological aging. Nevertheless, these measures are acknowledged to be incomplete summaries of biological changes that occur with aging and to have technical limitations [57, 58]. Treatment effects on aspects of biological aging not captured by the DNAm measures are not included in effect estimates; measurement error due to technical limitations of DNAm assays may bias effect estimates towards the null. Treatment effect estimates may therefore represent a lower bound of the true impact of vegan or omnivore dietary intervention on biological aging.

A notable contribution of this study is the assessment of Epigenetic Biomarker Proxies (EBP), which were previously described [14]. Firstly, the notable consistency in significant decreases observed in both BMI-EBP and BMI-clinical values across diet types highlights the reproducibility of BMI metrics within the epigenetic context. Despite a slightly higher magnitude of change in BMI-clinical values, the parallel trends emphasize the reliability of BMI-EBPs as reflective markers of body mass index alterations. Secondly, six EBPs exhibited divergent alterations between the vegan and omnivore diets, shedding light on diet-specific impacts on the epigenome. Ergothioneine, indoleacetylglutamine, and creatinine demonstrated a noteworthy decrease in the vegan group but an increase in the omnivore group. Ergothioneine, a potent antioxidant guarding cells against oxidative stress, potentially decreased in the vegan diet due to reduced intake from sources like mushrooms and certain grains [44]. Indoleacetylglutamine, derived from tryptophan, showcased elevated levels in the omnivore diet and a decline in the vegan diet, possibly mirroring the distinct abundance of protein-rich foods in each diet. The analogous patterns in creatinine, a marker of muscle metabolism, might also be linked to variations in protein intake and muscle turnover between the two diets. Conversely, serine, 1-margaroyl-GPE(17:0), and 4-acetamidophenol saw a significant rise in the vegan group but a decrease in the omnivore group. Serine, a non-essential amino acid abundant in plant sources, such as soybeans and nuts, likely increased on the vegan diet due to elevated consumption. The opposite trends in 1-margaroyl-GPE(17:0), a relatively novel metabolite predicted to function as a glycerophospholipid involved in cellular membranes and signaling pathways, suggest diet-induced variations in membrane composition and function. 4-acetamidophenol, a derivative of paracetamol widely used in analgesic and antipyretic medications, may reflect increased usage in the vegan compared to the omnivore group. Further studies are needed to identify the health implications of these changes and whether specific dietary components are responsible for them. Thirdly, the analysis of the previously published EpiScores provided insights into the potential of DNA methylation markers for predicting complex physiological and behavioral traits influenced by diet [37]. While seven EpiScores showed small effect changes exclusively in the vegan group and six exhibited exclusive significance in the omnivore group, the failure to achieve the corrected *p*-value provides an avenue for further interrogation of their utility in interventional data. However, the EpiScores identified by the uncorrected *p*-value threshold do act as targets for further assessment in clinical and lab-based protein studies. Nevertheless, the significant changes in EBP values highlight the potential of DNA methylation-based surrogate markers in delineating diet-related impacts on complex traits. This underscores the necessity for further exploration to refine and validate these markers for their predictive utility.

Several metabolites EBPs exhibited noteworthy changes, providing insights into differences and commonalities of diet response between the two groups. Among the top markers showing significant alterations in the vegan group, C-reactive protein (CRP), deoxycholic acid glucuronide, and spermidine stood out. A decrease in predicted CRP levels suggests a potential reduction in systemic inflammation. Spermidine, a polyamine associated with various health benefits, demonstrated an increase, potentially indicating an increased intake of vegetables like soy, legumes, and mushrooms. Deoxycholic acid glucuronide, a bile acid metabolite, displayed a decrease, suggesting an expected potential reduction in bile acid metabolism in response to a reduced intake of animal fat. Additionally, the vegan group demonstrated significant changes in other markers, such as N-acetyl-cadaverine and carnitine. Whereas the elevated levels of N-acetyl-cadaverine decreased as expected, given that this marker is associated with amino acid fermentation in the gut, the increase in carnitine levels contradicts the anticipated decrease in response to a vegan diet, since carnitine is mainly derived from meat and dairy products [59].

Several metabolites exhibited significant decreases in both diet groups, pointing to shared metabolic responses across diverse dietary patterns. Both salicylate, a component found in various plant foods, and its metabolite salicyluric glucuronide, demonstrated a reduction in both groups potentially reflecting a decrease in salicylate rich food such as legumes (e.g., lentils, beans), vegetables (e.g., cauliflowers, pickled vegetables), and fruits (e.g., strawberries, plums, watermelons). Reductions in quinate, a compound derived from the metabolism of coffee polyphenols [60, 61] and 10-undecenoate (11:1n1), a fatty acid related to butter intake [62, 63], suggest potential reduction in coffee and butter intake, respectively. Interestingly, both groups exhibited a decrease in predicted body mass index (BMI), which is consistent with the decrease in BMI in both groups.

In the omnivore group, we observed several intriguing shifts in key metabolic markers. The increase in tryptophan and serotonin, a neurotransmitter synthesized from tryptophan, suggests potential impacts on mood regulation and other serotonin-mediated functions in response to increased intake of tryptophan-rich animal protein in the omnivore diet. Choline phosphate, a vital component in cell membrane structure, exhibited an increase, hinting at increased dietary intake from meat, fish, and eggs. Indolebutyrate, a microbial metabolite, displayed an increase, suggesting potential shifts in gut microbial metabolism influenced by the diverse dietary components. Adenosine, a nucleoside that promotes sleep and reduces anxiety, exhibited an increase, indicating potential changes in endogenous metabolism on an omnivore diet [64]. These findings underscore the nuanced interplay of neurotransmitter synthesis, lipid metabolism, microbial activity, and purine metabolism associated with omnivorous dietary patterns.

Previous studies have suggested vegan diets associated with lower T2D risk [40, 41]. Interestingly, our investigation into T2D risk-associated methylation loci revealed that the vegan diet led to increased methylation in *ABCG1* and *PHOSPHO1*, which provided relatively conflicting results; increase in *ABCG1* indicates a reduced T2D risk, which is contradicted with the increase in *PHOSPHO1*, which indicates increased T2D risk. These results call for the need to develop disease-specific epigenetic predictors for T2D risk which go beyond single loci risk predictors, to potential multi-loci risk predictors exhibit significant association to disease risk.

Finally, the exploration of global DNA methylation patterns across the entire epigenome revealed significant differences between the vegan and omnivore cohorts, and identified 607 and 494 differentially methylated loci (DMLs) across the genome, respectively. Notably, the models accounted for potential confounding factors such as BMI, age, and sex, making it likely that these DMLs are more closely associated with the diet change. This comprehensive epigenome-wide analysis aligns with a growing body of literature examining the epigenetic effects of different dietary patterns [65–67]. When we analyzed each diet group independently, we observed 322 hypomethylated probes in the vegan diet and 185 in the omnivore diet. These CpG sites represent the epigenetic targets that changed during the trial, but independent of diet. However, to compare the evolution of each of the twin pairs, we compared the week 8 time point for those individuals in the vegan diet and those in the omnivore diet. This analysis unraveled 980 DMLs, with 317 demonstrating higher methylation in the vegan group and 663 in the omnivore group. Using the significant CpG sites from the twin-pair comparison at week 8, we performed an enrichment analysis to elucidate the biological relevance of these methylation patterns. Hypermethylated sites in vegans revealed enrichment of paracrine signaling, response to beta-amyloid, neuron apoptosis, and developmental processes. These findings imply that a vegan diet may influence pathways associated with cellular communication, neuroprotective mechanisms, and development. In contrast, hypermethylation in omnivores was linked to cell cycle regulation, genomic imprinting, cytosolic calcium ion transport, and cellular response to alcohol. This suggests that an omnivorous diet may impact pathways related to cell division, genetic regulation, cellular signaling, and responses to environmental stimuli. These insights contribute to a deeper understanding of how diet can impact the epigenome and, consequently, influence various aspects of cellular activity and health outcomes. Future investigations linking the epigenetic sites identified here in the context of gene expression may identify gene regulatory networks altered due to diet, further providing a molecular perspective in nutrition and diet.

Our exploratory longitudinal differential methylation analyses were focused on identifying candidate DNA methylation loci associated with 8 weeks of a vegan or herbivore diet. Hence, we utilized a more stringent *P* value cutoff of less than 0.001 which has been utilized by other EWAS studies [68, 69]. Our differential methylation analyses also controlled for twin structure and other potential confounding factors of age, sex, BMI, batch, immune cell composition, and accounted for the repeated measures by considering twin pairs as a random effect. However, this approach may have identified DML by chance and is a limitation of this approach compared to more stringent false discovery rate correction of all CpG loci. Due to the limited sample size of our study, when *p*-values were adjusted for multiple correction using the false-discovery rate method as has been utilized in large epigenome-wide association studies, in all three comparisons, this approach appeared too conservative as no DMLs were identified. Future studies are needed to validate DML associated with vegan and herbivore diets.

It is crucial to acknowledge that the observed epigenetic age and biomarker differences between the vegan and omnivore groups may be predominantly attributed to the variations in weight loss rather than solely reflecting the distinct dietary compositions. Throughout the “Food Delivery” phase, the vegan group consumed ~ 200 calories less per day than their omnivorous counterparts, resulting in an average weight loss of 2 kg greater than the omnivore group by the end of the 8-week intervention. Extensive population studies and Mendelian randomization analyses have underscored the impact of BMI changes on inducing epigenetic alterations linked to metabolic health [70, 71]. However, it should be noted that while we saw significant decreases in both clinical-BMI and EBP-BMI values, only the vegan cohort exhibited significant reductions in epigenetic age. This calls for a nuanced interpretation of our findings and emphasizes the need for future investigations to disentangle the complex interrelationships between dietary factors, weight dynamics, and epigenetic modifications.

While our study provides valuable insights into the short-term effects of weight loss on two different diets on epigenetic markers, it is important to acknowledge that the long-term impact of a vegan diet on epigenetic processes may carry adverse effects in the absence of sufficient intake of crucial vitamins and nutrients essential for supporting these intricate molecular reactions. In particular, all vegans and a substantial portion of vegetarians, if not supplemented, are at risk of developing vitamin B12 deficiency, resulting in elevated levels of homocysteine—an established marker of dysfunctional methylation associated with increased cardiovascular risk, including coronary artery disease (CAD) and heightened stroke susceptibility [72–74]. Vitamin B12 deficiency has been implicated in disease-related epigenetic alterations in both animals and humans [75–78]. In our cohort, the vegan group exhibited a lower intake of vitamin B12, although serum vitamin B12 levels did not demonstrate statistical differences compared to omnivores at the 8-week mark, likely due to preserved stores [21]. It is crucial to emphasize that long-term adherence to vegan diets typically necessitates vitamin B12 supplementation to mitigate the risk of deficiency and its consequential impact on epigenetic processes. Furthermore, the vegan cohort exhibited lower caloric intake, consumed less saturated fats, more polyunsaturated fats, and more fiber than the omnivorous group, suggesting these as the potential drivers of age reductions, rather than the vegan diet only [21]. This highlights the imperative role of nutritional considerations in optimizing the health outcomes associated with plant-based dietary choices. Within the context of these limitations, our findings have implications for future geroscience research. Aging biology research has identified multiple therapies with the potential to improve healthy lifespan in humans. A barrier to advancing the translation of these therapies through human trials is that intervention studies run for months or years, but human aging takes decades to cause disease [79–81].

We also acknowledge the potential for differences in behavior and lifestyle factors which may have impacted the study findings here. As previously described (21, online Supplement 2, eAppendix), majority of factors which may alter methylation changes were controlled for among the individuals who participated in the trial: routine dietary checks throughout the duration of the trail, a fixed checklist for diet adherence, and assessment of diet adherence at the end of each 4-week phase. In one sensitivity analysis which identified non-normal changes in a group of twins featured in a documentary compared to the rest of the group. These analyses identified differences in TMAO levels in a set of twins which were removed from the study, indicating a potential confounding factor of non-adherence to the preset diet which was corrected for. A subset of twins (*N* = 4) contributed to the filming of a documentary and thus were encouraged to exercise more, which may affect caloric outputs and thus epigenetic changes [82]. While the current analyses accounted for the large effects using a pair-wise and random effect statistical design, minor effects in the cross-sectional analyses may not have been accounted for.

## Conclusions

In this epigenetic analysis of an initial randomized clinical trial, we observed significant changes using epigenetic age clocks among healthy identical twins, suggesting short-term advantageous aging benefits for a calorie-restricted vegan diet compared to an omnivorous diet. The use of EBPs in this study showcases the potential of epigenetic testing to provide personalized insights into the impact of nutrition on cellular aging, enabling targeted dietary interventions to optimize health and well-being. Differential methylation analysis of diet type identified methylation changes unique to each diet implementation, potentially representing methylation markers of diet. However, it is still uncertain whether the observed benefits may be primarily due to greater weight loss in the vegan group; thus long-term effects of unsupplemented vegan diets on epigenetic processes require further investigation. Future research utilizing a long-term, well-controlled study design will further highlight the complex relationships between diet, epigenetics, and health outcomes such as weight loss, while emphasizing the importance of proper nutrient supplementation in vegan diets.

### Supplementary Information

Additional file 1: Table S1. EpiScore analysis between baseline and 8-week test in the Stanford TWINs trial.The first column reports the EpiScore that was assessed, followed by the unadjusted*p*-value, and the direction of difference of the residual values between Week 8 from Week 0 for the Vegan (columns 2 and 3), and omnivore samples (columns 4 and 5). The final two columns show adjusted *p*-values for the vegan (column 6) and omnivore analyses (column 7). The statistical test run here was the Wilcoxon-rank sum test. The direction of change is represented as a + (representing a higher value at Week 8 relative to Week 0) or a - (representing a lower value at Week 8 relative to Week 0). Abbreviations: NS = not significant.

Additional file 2: Table S2. Epigenetic Biomarker Proxy (EBP) analysis between baseline and 8-week test in the Stanford TWINs trial. The first column reports the EBP that was assessed, followed by the unadjusted *p*-value, and the direction of difference of the residual values between Week 8 from Week 0 for the Vegan (columns 2 and 3), and omnivore samples (columns 4 and 5). The final two columns show adjusted *p*-values for the vegan (column 6) and omnivore analyses (column 7). The statistical test run here was the Wilcoxon-rank sum test. The direction of change is represented as a + (representing a higher value at Week 8 relative to Week 0) or a - (representing a lower value at Week 8 relative to Week 0). Abbreviations: NS = not significant. 

Additional file 3: Table S3. Excel file contains the significant results for the differential methylation analysis results from the EWAS time point analysis of Week 8 vs Week 0 of the Vegan diet. Column headers of each sheet are listed as follows: Column A represents the CpGs identified; Column B shows the log fold change of the m-value between Week 0 vs. Week 8 for each timepoint comparison, in which positive values are higher methylation at week 8 relative to week 0; Column C shows the average M-value for the CpG; Column D reports the t-statistic; Column E reports the unadjusted *p*-value; Column F reports the false-discovery rate (FDR) corrected *p*-value; Column G reports the B value outputted from limma; and Column H reports the gene ID overlapping the specific CpG loci. Table S4. Excel file contains the significant results for the differential methylation analysis results from the EWAS time-point analysis of Week 8 vs Week 0 of the Omnivore diet. Column headers of each sheet are listed as follows: Column A represents the CpGs identified; Column B shows the log fold change of the m-value between Week 0 vs. Week 8 for each timepoint comparison, in which positive values are higher methylation at week 8 relative to week 0; Column C shows the average M-value for the CpG; Column D reports the t-statistic; Column E reports the unadjusted *p*-value; Column F reports the false-discovery rate (FDR) corrected *p*-value; Column G reports the B value outputted from limma; and Column H reports the gene ID overlapping the specific CpG loci. Table S5. Excel file contains the significant results for the differential methylation analysis results from the EWAS time-point analysis of the Week 8 Vegan compared to the Week 8 of the Omnivore diet. Column headers of each sheet are listed as follows: Column A represents the CpGs identified; Column B shows the log fold change of the m-value between the Vegan vs. Omnivore at Week 8, in which positive values are higher methylation in the vegans relative to omnivores; Column C shows the average M-value for the CpG; Column D reports the t-statistic; Column E reports the unadjusted *p*-value; Column F reports the false-discovery rate (FDR) corrected *p*-value; Column G reports the B value outputted from limma; and Column H reports the gene ID overlapping the specific CpG loci. Table S6. Excel file contains the significant results for the differential methylation analysis results from the EWAS time-point analysis of the Week 0 Vegan compared to the Week 0 of the Omnivore diet. Column headers of each sheet are listed as follows: Column A represents the CpGs identified; Column B shows the log fold change of the m-value between the Vegan vs. Omnivore at Week 8, in which positive values are higher methylation in the vegans relative to omnivores; Column C shows the average M-value for the CpG; Column D reports the t-statistic; Column E reports the unadjusted *p*-value; Column F reports the false-discovery rate (FDR) corrected *p*-value; Column G reports the B value outputted from limma; and Column H reports the gene ID overlapping the specific CpG loci. 

Additional file 4: Table S7. GREAT results for DMLs hypermethylated in Vegan samples at 8 weeks, compared to Omnivore samples. Columns presented are as follows: Column A represents the GO ID of the term identified; Column B exhibits the name of the GO ID; Column C exhibits the total number of genes matched from the list of CpGs; Column D represents the hypergeometric coefficient expected; Column E represents the hypergeometric coefficient observed from the CPG set;  Column G represents the fold enrichment of the hypergeometric value; Column G represents the raw hypergeometric test*p*-value; Column H represents the adjusted hypergeometric test *p*-value (BH); and Column I represents the class of gene ontology (GO) term - Molecular Function (MF), Biological Process (BP), and Cellular Component (CC). Table S8. GREAT results for DMLs hypomethylated in Vegan samples at 8 weeks, compared to Omnivore samples. Columns presented are as follows: Column A represents the GO ID of the term identified; Column B exhibits the name of the GO ID; Column C exhibits the total number of genes matched from the list of CpGs; Column D represents the hypergeometric coefficient expected; Column E represents the hypergeometric coefficient observed from the CPG set;  Column G represents the fold enrichment of the hypergeometric value; Column G represents the raw hypergeometric test *p*-value; Column H represents the adjusted hypergeometric test *p*-value (BH); and Column I represents the class of gene ontology (GO) term - Molecular Function (MF), Biological Process (BP), and Cellular Component (CC).

## Data Availability

The data that support the findings of this study are not publicly available due to protection of patient data in accordance to maintaining HIPAA compliance. However, the corresponding authors can provide the data upon reasonable request after signing a Data Use Agreement.
